# From Farm to Table and Back Again: Circular Valorization of Biomass Ash and Sewage Sludge into Sustainable Material Blends

**DOI:** 10.3390/ma19081552

**Published:** 2026-04-13

**Authors:** Ekaterina Serafimova, Vilma Petkova, Veneta Petkova

**Affiliations:** 1 Department of Environmental Engineering, University of Chemical Technology and Metallurgy, 8 Kliment Ohridski Blvd., 1756 Sofia, Bulgaria; gvenddy@gmail.com; 2 Institute of Mineralogy and Crystallography, Bulgarian Academy of Sciences, Acad. G. Bonchev Str., Bl. 107, 1113 Sofia, Bulgaria; vilmapetkova@gmail.com

**Keywords:** biomass ash, wastewater sludge, zeolite, pozzolanic activity, mineral phases, circular economy, waste valorization

## Abstract

In the era of increasing generation of various waste streams, the possibility of utilizing them as secondary resources is of utmost importance and fully corresponds to the goals of the circular economy. Industrial residues from the pulp and paper industry, such as biomass combustion ash (FARP) and sludge from industrial wastewater treatment (PPWS), together with natural zeolite as a modifying additive, represent valuable sources enabling their integrated valorization. The present study aims to investigate the potential for their reuse through the development of sustainable material blends. A comprehensive analysis of the chemical composition and morphology of the obtained mixtures was carried out using inductively coupled plasma optical emission spectroscopy (ICP-OES), X-ray diffraction (XRD), and scanning electron microscopy (SEM). The results indicate a tendency for the formation of mineral matrices dominated by calcium–sulfur–oxygen (Ca–S–O) phases, with the presence of calcium sulfate and aluminosilicate structures. The blends are associated with the formation of stable crystalline structures exhibiting potential pozzolanic activity. In this way, carbon is captured and fixed in a stable mineral form. The obtained results suggest the potential of these blends for use in low-carbon systems focused on waste valorization and carbon retention. The materials may be suitable for applications in construction, soil remediation, and environmental technologies, contributing to closing the resource loop “from farm to table and back again”.

## 1. Introduction

Climate change impacts are intensifying. As a reaction, the European Union (EU) have harmonized its policies and actions to lay out frameworks and objectives for addressing this effectively. To achieve these results with guaranteed economic efficiency, environmental resilience, and social acceptability, it is necessary to introduce integrated practices for resource utilization of waste generated by industry. The EU aims to lower the environmental footprint of its food system and increase its resilience. It also aims at ensuring food security in the face of climate and biodiversity pressures, as well as facilitating the transition to sustainable “farm-to-table” systems. The generation of industrial waste from the pulp and paper industry remains a challenge in certain sectors worldwide [1]. The challenges are related to biomass ash from various combustion boilers and wastewater sludge from production activities. Working with waste materials follows contemporary ideas of sustainability. They highlight the multifunctional function of resource systems on environmental protection and sustainable development [2]. Increasingly, these materials are mentioned in the scientific literature as valuable secondary resources [3,4,5]. Within the concept and objectives of the circular economy, these materials are evaluated due to their mineral and chemical composition and potential for reuse in sustainable material applications.

The pulp and paper industry has an increasing share of the global economy, despite digitalization, as other technologies for its application are also developing. On the other hand, the emerging trends of the circular economy set ambitious goals in the production sector for the full utilization of raw materials and waste, which in turn leads to different quantities and types of ash. Fly ash from boilers in the pulp and paper industry is treated as non-hazardous industrial waste and is landfilled, which leads to costs as well as occupation of land areas that could be used for other purposes. For this reason, alternatives are being sought for environmentally efficient mechanisms to find applications for this ash, despite its sometimes-variable composition. Scientific articles present various alternatives for reuse, such as composting sludge with ash, using ash for neutralization of acidic soils, as fertilizer, soil amendment, and others. Biomass ash has been widely investigated and is recognized as a calcium-rich material useful for soil amendment, stabilization and as an additive in cementitious systems due to its alkalinity and mineral compositions [6,7,8]. On the one hand, sewage sludge has also been considered as a second valuable resource mainly due to its organic matter and mineral constituents which can be used in agriculture and construction industry [9,10]. The added value of this type of waste is multifunctional and can lead to integrated and sustainable engineering applications. Ash is utilized in various applications. These uses include supplementary cementitious material in concrete, aggregate for road construction, soil stabilization and adsorption of toxic heavy metals [11,12,13,14,15,16,17,18].

Sludge from industrial wastewater treatment plants contains an organic fraction, along with mineral components such as macro- and microelements, which participate in the main biogeochemical cycles of substances and can contribute to stabilization of materials and reduction in the use of primary resources [19,20,21]. There are numerous developed and established methods for the treatment of sludge generated from wastewater treatment, such as landfilling, aerobic and anaerobic stabilization, application in agriculture, thermal treatment with energy recovery, and use in construction materials and environmental technologies [5,19,20]. The legislative requirements are getting stricter, especially in terms of landfilling and concentrations of components in the sludge allowed. This makes it difficult to use and dispose of. Therefore, the effective management of this sludge, from both economic and environmental perspectives, and the selection of an appropriate treatment method is a labour-intensive process. Similarly, biomass combustion ash is rich in calcium, silicon, and other mineral components, making it suitable for reuse as a supplementary material in sustainable material systems [22,23,24,25]. The chemical composition of biomass ash may vary depending on the type of biomass and combustion conditions, but calcium often represents the dominant component, providing potential for the development and stabilization of mineral phases [26,27].

From the perspective of materials science, the formation of systems capable of developing stable mineral binding phases is of significant interest. One of the important materials that can be used in such systems is calcium. It may also generates calcium silicate hydrates (C–S–H) and calcium aluminosilicate hydrates (C–A–S–H) in environments rich in aluminium and silicon. These phases typically are structurally stable and have good binding [11,12,27,28,29]. The presence of hydrate phases and pozzolanic material (fly ash, silica fume, slag) increases the strength of the internal structure by providing mechanical strength and long-term durability. When industrial by-products containing such phases are used, they can serve as a substitute resource for the formation of such mineral structures, contributing to the stability of materials and their long-term operational reliability [16,17,18].

Besides the general one of characterizing the materials, it is necessary to address them individually from their physicochemical properties in order to better understand what happens when they are together in a system. Sludge from industrial wastewater treatment contains high amounts of organic matters and four different types of mineral components including carbonate phases, which play important roles on physicochemical interaction as well as mechanical properties of products [5,19,20,21]. In contrast, biomass ashes consist predominantly of reactive oxides and calcium-rich mineral phases capable of pozzolanic reactivity leading to the formation of cementitious compounds [22,23,24,25,27]. Zeolite, a crystalline aluminosilicate material, offers a porous structure with high cation exchange capacity, which aids in ion immobilization and improves structural stability [30,31]. Natural zeolites are widely recognized for their ion-exchange capacity, adsorption properties, and pozzolanic activity, which make them suitable for use in environmental and construction applications [29,30,31]. The knowledge of the behaviour of these materials separately is crucial for understanding their influence in mixed systems and their contributions to soil binding mechanisms, as well as for stable mineral phases formation [11,16,17,18].

The integrated approach for the utilization of two types of waste materials, such as biomass ash and wastewater sludge from the same industrial production, along with the addition of zeolite, leads to the formation of new structures with specific functional properties. Biomass ash provides a calcium-rich mineral matrix, wastewater sludge contributes reactive and amorphous components, and zeolite provides stable aluminosilicate phases. Mixtures structured in this way, in different proportions, would have the potential to form materials that support sustainable resource utilization processes.

The environmental benefits and reuse of materials contribute to waste reduction, eliminate the need for new natural resources, and thereby reduce greenhouse gas emissions associated with new production activities for resource extraction. The use of calcium, in turn, supports the stabilization of carbon in mineral forms and contributes to carbon retention, which positively affects environmental sustainability. The transformation of waste into stable mineral composites corresponds to the principles of the circular economy and supports the development of low-carbon material solutions [12,14,19].

Despite the considerable scientific interest in recent years in methods and approaches for the utilization and reuse of various materials, there are certain scientific gaps to which this article can add value, namely the use of zeolite within a specific concentration range and acid activation. Another specific feature of biomass ash is that its chemical and mineral composition varies depending on the raw material used during combustion, whether it is only wood or includes other plant residues, as well as the climatic, edaphic, and orographic conditions under which they developed. This may alter the properties and characteristics of the waste. Different mixture ratios would provide new knowledge on structural evolution and trends through integrated chemical, mineralogical, and microstructural analysis.

The present study aims to investigate the chemical, mineralogical, and structural characteristics of mixtures obtained based on biomass ash, industrial wastewater sludge from the pulp and paper industry, and zeolite. Based on the obtained mixtures, their potential to form stable mineral structures suitable for sustainable material applications related to soil improvement and pozzolanic activity of the substances will be evaluated. The study is oriented toward understanding the compositional changes and trends according to different mixture ratios, as well as their development at the phase and structural levels. Based on the obtained results, the merits of the materials and their contribution to the development of strategies for sustainable resource utilization within the framework of the circular economy will be assessed.

It is hypothesized that the combination of biomass ash and sewage sludge, followed by acid activation and zeolite modification, promotes mineral transformation and leads to the formation of stable calcium–sulfate–aluminosilicate structures capable of stabilizing carbon and enhancing the structural and functional properties of the resulting material blends.

## 2. Materials and Methods

### 2.1. Raw Materials

As a raw material, sludge from the treatment plant of “Svilosa AD”, Svishtov, Bulgaria, from the pulp and paper industry was used in PPWS with 8% moisture content. Environmental pollution, together with the costs for utilization and reclamation of sludge storage landfills, represents a serious environmental problem for the region. The reuse of nutrients from wastewater treatment plant sludge in soil has been proven to be beneficial, favorable, and leads to improvement of soil condition and land productivity. Dewatered wastewater sludge contains valuable resources and minerals that are beneficial for soil and plants. There is an increasing trend in sludge utilization, as it essentially represents organic material, water, organic matter, and nutrients. In order to properly characterize the importance of raw materials in a perfect system, it is important to emphasize their main physicochemical parameters. In order to properly characterize the importance of raw materials in a perfect system, it is important to emphasize their main physicochemical parameters.

The high content of calcium oxides, hydroxides and carbonates gives biomass ash (FARP) a strongly alkaline character. The alkaline nature of the solution gives it buffering capacity and determines its chemical reactivity in mineral transformation processes. Due to its high calcium content, it is a major precursor for calcium-based mineral phases such as calcium silicate hydrates (C–S–H) or calcium aluminosilicate hydrates (C–A–S–H) that are critical for structural development.

On the other hand, wastewater sludge (PPWS) possesses a predominantly amorphous structure and a high fraction of organic matter. With low degrees of crystallinity, sludge is thus more reactive and acts as a reactive matrix that enhances mineral–mineral interactions, promoting organo-mineral structure formation.

H_2_SO_4_ (sulfuric acid, 95–98%, analytical grade, Sigma-Aldrich, St. Louis, MO, USA) acts as an activating agent. Because of it has a strong acidity and promotes the dissolution of carbonate phases, such as CaCO_3_, and also induces sulfation processes that promote the formation of calcium sulfate phases. The activation alters the mineral composition with a view toward activating phase transformation in the system.

Clinoptilolite type natural zeolite from the Beli Plast deposit, Bulgaria has developed porous structures and high Si/Al ratios as well as a very high cation exchange capacity (CEC). These properties establish its ability to sorption capacity and pozzolan reaction participation. The studied system uses zeolite as a structural modifier and nucleation site providing active sites for the formation of stable aluminosilicate phases, linking them together and producing mortars with increased overall stability of the mineral matrix.

This paper evaluates the zeolite which is a natural clinoptilolite-type aluminosilicate with high specific surface area, well-developed porous structure and high cation exchange capacity. The fact that it is rich in silica (Si) and alumina (Al), thus capable of pozzolanic behaviour, also aids in the build-up of calcium aluminosilicate hydrate (C–A–S–H) phases. Moreover, zeolite has strong sorption properties that allow for nutrient retention and the immobilization of potentially toxic elements in a mineral matrix.

The ash used was obtained as a result of the operation of a biomass boiler at a pulp and paper production plant in Svilosa AD, Svishtov, Bulgaria. The combustion temperature and heat of combustion were approximately 1000–1100 °C. The combustion temperature itself leads to formation of ash and slag resulting from incomplete combustion. The FAPR was collected from a multicyclone dust collector integrated into a steam generator system (manufactured by “Invest Stroy Montazh” Ltd., Svishtov, Bulgaria), during the combustion of waste wood and plant residues. The ash has a distinctly alkaline character, which defines it as a suitable material for the treatment of acidic soils. A favorable prerequisite is also that it contains certain quantities of nutrient elements in proportions similar to those present in the original wood and/or plant biomass. The generated quantities are approximately 7 kg/t or 800 t/year.

Sampling was performed in accordance with EN 14899:2005 (Characterization of waste—Sampling of waste materials—Framework for the preparation and application of a Sampling Plan) [32], using 30 grab samples collected over a period of 10–15 days, twice daily from different locations.

**Figure 1:**
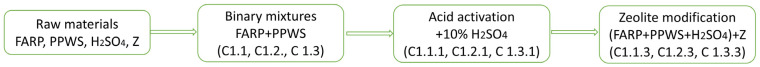
Schematic preparation and development pathway of the mixtures.

The preparation of the mixtures involved sequential mixing of the raw materials in predetermined mass ratios. Initially, PPWS and FARP were homogenized under dry conditions. Subsequently, sulfuric acid was gradually added under continuous stirring to ensure uniform distribution and controlled sulfation. The mixtures were aged for 24 h at room temperature, followed by drying at 105 °C until constant mass. In the final stage, zeolite was incorporated into the activated mixtures and homogenized to obtain the final composite materials. The sample preparation procedure is illustrated in Figure 1, and the mixture ratios are presented in Table 1.

### 2.2. Justification of the Mixture Ratios

In the mass fractions of mixtures C 1.1, C 1.2, and C 1.3, the aim was to cover three ranges of FARP: low (C 1.1), medium (C 1.3), and high (C 1.2), with the content varying from 25 to 42 wt%. FARP is an important component, as it has the ability to build a mineral skeleton that contributes to variations in porosity and water demand. On the other hand, it is an important source of mineral compounds, and its buffering capacity is necessary for soil amendments, from the point of view of its potential to influence the active reaction. From the perspective of pozzolanic activity, the mineral composition of FARP would provide a calcium-based structural framework and nucleation sites, supporting the formation of binding phases upon activation.

PPWS is associated with the addition of elements and compounds that support processes related to stability and sorption. Due to its amorphous structure, it would have an advantage in terms of potential reactivity and a synergistic effect with FARP.

Sulfuric acid was applied at a constant degree of activation to allow better controlled comparison of its effect on the main raw materials. This dosage was selected based on previous experience [33], where good potential was established for surface phase modification, sulfation, and the transition of important nutrient elements into a bioavailable form in the soil solution. Full neutralization was not the objective; rather, the aim was to enable variation under conditions of partial neutralization. Its role in pozzolanic activity is related to its potential to dissolve and modify carbonate phases. This leads to changes in the availability of reaction centers and to the formation of sulfate phases that influence nucleation.

Zeolite was also added at a constant proportion of approximately 7%, which would provide a sufficiently effective level of modification through sorption surface activation. By its nature, it acts as a pozzolan due to its aluminosilicate composition. It is expected to provide nucleation sites and improve porosity, while its constant proportion as an additive in the mixtures would allow tracking of the influence of the pozzolanic additive on different calcium ratios.

FARP and PPWS differ in their structural and physicochemical composition. Therefore, by varying their ratios, the effort was directed toward structural variation and modification, with one case favoring the amorphous fraction and the other the crystalline fraction. These transformations aim at achieving different degrees of neutralization under controlled sulfation conditions. This also makes it possible to track calcium availability for C–S–H/C–A–S–H formation and to evaluate the potential of the amorphous reactive matrix as a pozzolanic component.

### 2.3. Methods

The C, H, and S content was determined using an Elemental Analyzer FlashSmart EA CHNS (Thermo Scientific™, Bremen, Germany) in the accredited laboratory. Calibration for sulfur was carried out with certified pure substances, and the resulting calibration uncertainty ranged from 16% at low to 4% at high sulfur concentrations. Additional elemental analyses, including the determination of heavy metals (Pb, Cd, Cr, Ni, Zn, Cu, As) and other major and minor elements, were performed using an ICP-OES spectrometer SPECTRO ARCOS (SPECTRO Analytical Instruments GmbH, Kleve, Germany) in the accredited laboratory. The calibration uncertainty for ICP-OES ranged from 15% to 3%, depending on the element concentration. The measured elemental concentrations were subsequently recalculated into their corresponding oxide forms. The instrument was calibrated with certified reference materials, all of which are metrologically traceable.

Phase composition was determined using a PANalytical Empyrean X-ray diffractometer (Malvern Panalytical B.V., Almelo, The Netherlands) in the Laboratory for X-ray Diffraction Methods and Computer Tomography. The measurements were performed using a θ–θ goniometer configuration with a flat sample stage. Cu Kα radiation was used (K-α1 = 1.54060 Å, K-α2 = 1.54443 Å; K-β = 1.39225 Å) at generator settings of 40 kV and 30 mA. Data were collected in the 2θ range from 5.03° to 79.97°, with a step size of 0.053° 2θ and a scan time of 93.33 s per step, using continuous scanning in PSD mode (PSD length 3.35° 2θ). A fixed divergence slit of 0.5° was applied, with a goniometer radius of 240 mm. Measurements were carried out at 25 °C, without sample spinning or incident-beam monochromator. Fine calibration of the instrument was performed, with a 2θ offset of −0.0004°.

The measurements of the infrared spectra were performed on an FT-IR spectrometer, Varian 660-IR, Varian Inc., Palo Alto, CA, USA, 2009, covering the range of 400–4000 cm^−1^. The measurements were carried out at a spectral resolution of 4 cm^−1^. We prepared the specimens as a pellet of low-dispersed KBr and powdered the prepared mixture. The obtained mixture was pressed into a pellet with a diameter of 13 mm under a pressure of approximately 10 MPa. Before each measurement, a background spectrum of a pure KBr pellet was recorded under identical conditions. The obtained spectra were processed by baseline correction and normalization using the instrument software. All measurements were conducted at room temperature (25 ± 1 °C). The wavenumber precision is 0.003 cm^−1^, and the wavenumber accuracy is 0.005 cm^−1^ at 2200 cm^−1^. The verification of the wavenumber scale was performed periodically using a polystyrene standard reference material (SRM) with metrological traceability to NIST.

The surface morphology of the samples was studied with scanning electron microscopy (SEM) using a Zeiss Evo 10 microscope (Carl Zeiss Microscopy, Oberkochen, Germany). The images were taken in secondary electrons mode with an accelerating voltage of 15 keV, and no conductive coating on the surface. The chemical composition of the samples was studied using the Oxford Ultim Max 40 electron dispersive spectroscopy (EDS) probe (Oxford Instruments, Abingdon, UK). The results were compiled with AZtec software (version 6.1 HF4). The detection limit of EDS was approximately 0.1–0.5 wt.%. The system was calibrated using factory standards.

## 3. Results

### 3.1. Elemental and Chemical Composition

#### 3.1.1. Characterization of Raw Materials

The chemical composition of the materials determines the distribution of substances in the mixtures and their corresponding applications. Table 2 presents the results of the performed chemical analyses.

Calcium is present in the highest concentration in FARP (30.48%), followed by its presence in zeolite (2.48%), and a minimal amount of 0.29% in PPWS. The trend for sulfur is the opposite. PPWS is an organically dominated raw material. Zeolite is associated with the addition of aluminum in the mixtures for the formation of an aluminosilicate component, with 4.02%, followed by FARP with 1.2%.

Each of the raw materials contains trace elements, with FARP contributing the highest concentrations of trace elements, and during mixing, a trend toward their concentration reduction is intended. Fe, K, and P are present in the highest amounts in FARP, followed by zeolite. For Na, the amount of 3175 mg/kg is in zeolite, while the lowest is in PPWS at 984 mg/kg. Based on the results presented above, FARP is the leading raw material in the mixtures.

#### 3.1.2. Characterization of Mixtures

The chemical and elemental composition of the prepared mixtures was analyzed to detect major and trace elements. The results are presented in Table 3 and Table 4. The effect of compositional variations and activation on their overall chemical characteristics is key to understanding the role of the mixtures and the chemistry of the processes for their application as new material structures.

The results of the chemical and elemental analysis were considered as triplets relative to the binary mixtures, acid activation mixtures, and zeolite modification mixtures, according to Figure 1, as well as in relation to the key elements for their potential application as soil amendments and materials exhibiting pozzolanic behavior. In the base mixtures C 1.1, C 1.2, and C 1.3, a very low percentage of sulfur (approximately 0.05%) was observed, but with a high concentration of calcium (approximately 30–33%), which acts as a donor for controlling binding phases.

With the addition of sulfuric acid, sulfur increased from 3% to 8.7%, at the expense of a decrease in calcium content (13.2–24.4%). In the zeolite modification mixtures, sulfur remained stable, but calcium decreased, or more precisely, was redistributed.

From the perspective of the molar Ca/S ratio, it decreased in the C 1.1.3, C 1.2.3, and C 1.3.3 series, which may be associated with the fact that during activation, reactive calcium is redirected toward the formation of a Ca–S–O matrix.

Aluminum has an important contribution to pozzolanic activity and the potential formation of C–A–S–H structures. The observed trends in the mixtures are as follows: in C 1.1, C 1.2, and C 1.3, aluminum is present at the highest percentages (1.2–12.4%); in C 1.1.1, C 1.2.1, and C 1.3.1, it decreases (0.70–0.91%); and partial recovery is observed in the C 1.1.3, C 1.2.3, and C 1.3.3 series, due to the contribution of zeolite. The Al/Ca ratio is a structural indicator and reflects the transition from a calcium-dominant system (Ca–S–O) to an aluminosilicate-controlled system (C–A–S–H). In the base series, the Al/Ca ratio is approximately 0.04, indicating an excess of calcium, where Ca–S–O phases dominate and aluminosilicates do not significantly participate. In the modification series, the relative share of aluminum increases and calcium no longer dominates, suggesting a structure closer to that required for a C–A–S–H matrix. Therefore, these mixtures were designed to reduce calcium availability, enabling the formation of CaSO_4_ and CaCO_3_, while retaining sufficient calcium to participate in a hydrated aluminosilicate network.

Mg and Fe have a secondary role in matrix stability. They are present in the highest percentages in the base mixtures, decrease after activation, and reach the lowest concentrations in the zeolite-modified mixtures. Na and K are associated with alkalinity and the solubility of the aluminosilicate phase, and their decreasing trend after activation and stabilization may be related to redistribution within the solid matrix.

Phosphorus, in turn, competes with calcium, and its redistribution is monitored during activation and zeolite addition. It decreases systematically across all series and may indicate incorporation into calcium-containing phases, influencing reactive calcium availability and supporting C–A–S–H formation.

From the perspective of potential soil amendment applications, the macro elements (Ca, P, K, Mg) are present at the highest levels in the base mixtures, with a decreasing trend after activation and modification. Calcium content in mixtures C 1.1, C 1.2, and C 1.3 remain significantly high even after activation, despite concentration decreases, indicating potential buffering capacity. Phosphorus is present in the range of 0.25–0.56%, which may contribute to nutrient supply. Potassium (0.78–1.13%) and magnesium (0.52–0.88%) support discussion of the mixtures’ potential as supplementary nutrient sources. Sodium content remains within moderate limits and does not indicate an immediate risk of soil salinization.

The concentrations of heavy metals were also calculated and analyzed, which is an important component for their further application. In the mixtures, a decreasing trend was observed, following the same sequence from base mixtures to activated mixtures, with the lowest concentrations in the stabilized mixtures.

The purpose of conducting the elemental analysis was to monitor the organic fraction and carbon content, which may react and participate in stabilization, mineralization, and restructuring processes. The results are illustrated in Table 4.

From the results of the elemental analysis, trends of decreasing carbon content during activation and subsequent increase during stabilization in the zeolite-modified mixtures can again be observed. Nitrogen shows very low fluctuations in all mixtures, which is related to the fact that it is not affected by acid degradation and remains stabilized within the matrix. Hydrogen does not follow a specific trend. The highest value is observed in C 1.2.1 (1.55%), and the lowest in C 1.3.1 (0.89%). The C:N ratio ranges approximately between 36 and 57 atomic ratios, indicating the presence of a stable carbon component. The C:H ratio varies between 0.50 and 0.95, with a decreasing trend from base mixtures through stabilized to activated mixtures, which is an indicator of structural transformations. The C:S ratio reflects the degree of sulfation in the mixtures, as observed in the results, where C:S in mixtures C 1.1, C 1.2, and C 1.3 is 50–240 times higher compared to the activated mixtures. This serves as a marker of chemical transformation.

### 3.2. Phase Composition and Mineralogical Development

The phase composition was determined by XRD using reference databases (RRUFF and COD) [34,35,36], confirming the potential reactivity of the wastes.

#### 3.2.1. XRD of Raw Materials

**Figure 2:**
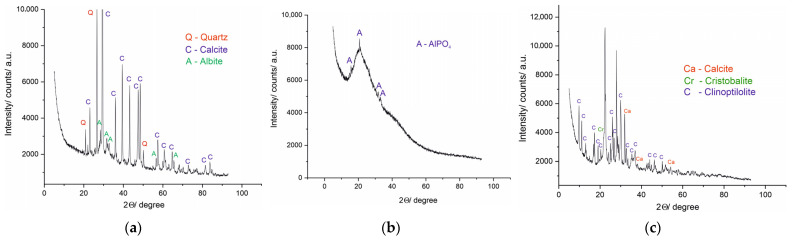
(**a**) XRD of FARP; (**b**) XRD of PPWS; (**c**) XRD of natural clinoptilolite.

In the crystalline mineral framework of FARP, calcite and quartz dominate as stable silica phases, along with albite (Figure 2a). Calcite indicates the presence of readily available calcium in the ash. Zeolite confirms its expected aluminosilicate framework and reactive aluminosilicate source, providing potential nucleation centers that support the formation of binding matrices (Figure 2c). PPWS is characterized by low crystallinity, expressed by the presence of an aluminum phosphate peak (Figure 2b). The amorphous organic fraction in the waste represents a valuable resource for activation and subsequent mineral transformation. This complementary phase composition supports the hypothesis and objective of the study regarding the potential formation of an active matrix.

#### 3.2.2. XRD of Mixtures

**Figure 3:**
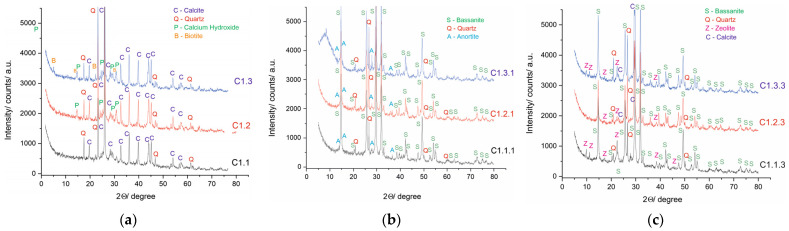
(**a**) XRD of binary mixtures; (**b**) XRD of acid-activated mixtures; (**c**) XRD of zeolite-modified mixtures.

XRD analysis was performed on three sets of mixtures to identify the phase composition of binary, acid activation, and zeolite modification mixtures. The results are presented in Figure 3a–c.

In the binary mixtures, the leading controlling parameter is calcium and its balance with Si + Al + P, as well as the phases formed from these components. In C 1.1, the dominant phases are CaCO_3_ (calcite) and SiO_2_ (quartz). In C 1.2, the dominant phases increase with the appearance of Ca(OH)_2_ (portlandite) and a complex Ca–P–(NO_3_)–hydrate phase, suggesting the possibility of phosphate/nitrate incorporation into calcium-containing hydrates. In C 1.3, CaCO_3_ (calcite), SiO_2_ (quartz), and Ca(OH)_2_ dominate again, along with K–Fe–Al silicate hydroxide, which represents a mica-clay mineral classified as a structural material. The differences between mixtures arise from variations in Ca/Si/Al ratios.

After treatment with sulfuric acid, phase transformation toward Ca–S–O structures is observed. The dominant phases in C 1.1.1 include calcium sulfate hydrate (CaSO_4_ × 0.5H_2_O), bassanite, quartz, and Na–Ca–Al silicate (CaAl_2_Si_2_O_8_ anortite or NaAlSi_3_O_8_ albite), confirming the intended rearrangement of parts of the Si/Al framework. In C 1.2.1 and C 1.3.1, bassanite and quartz phases are observed, with the sulfate phase becoming clearly dominant.

In the zeolite-modified activated mixtures, the clinoptilolite phase is present together with bassanite and quartz. In C 1.1.3, this contributes to sorption capacity. In C 1.3.3, bassanite, quartz, and calcite are present simultaneously, indicating the coexistence of sulfate and carbonate systems. In C 1.2.3, in addition to bassanite and quartz, complex aluminosilicates (Ca–Na–Al silicate (anortite or albite) and K–Mg–Fe silicate) are also present. This indicates a complex mineralogical structure of the mixture, resulting from the specific ratios of the raw materials.

### 3.3. FTIR Analysis

FTIR analysis of the mixtures is performed to identify functional groups and chemical bonds, allowing the interactions between components and the formation of new hydration products to be monitored. Through FTIR, changes in silicate, aluminate, and hydroxyl groups can be detected, providing information about the degree of pozzolanic reaction and the development of the cement matrix structure [37].

**Figure 4:**
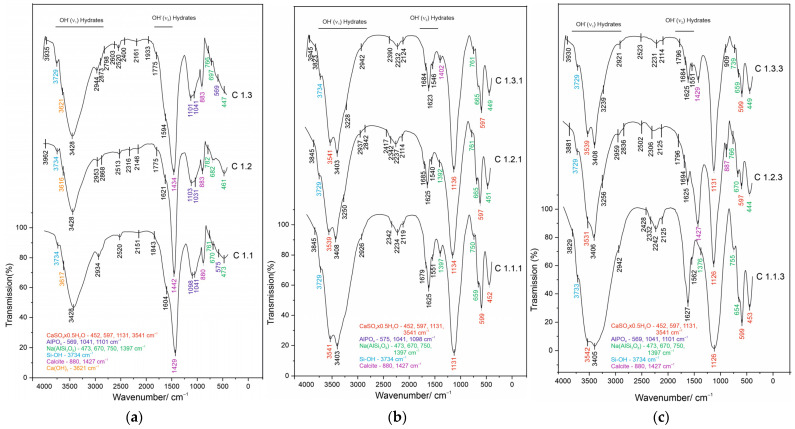
(**a**) FTIR spectra of binary mixtures; (**b**) FTIR spectra of acid-activated mixtures; (**c**) FTIR spectra of zeolite-modified mixtures.

The FTIR analysis results are given in Figure 4a–c.

The FTIR spectra of the non-activated mixtures (C1.1, C1.2, and C1.3) show a typical carbonate–silicate fingerprint. The bands in the region of ~1429–1442 cm^−1^, together with peaks around ~880–883 cm^−1^, indicate the presence of calcite, which is associated with the added ash. The characteristic band observed in all three mixtures in the range of ~3616–3621 cm^−1^ is associated with portlandite and the Ca–OH bond. The broad band at ~1031–1103 cm^−1^ and the absorption at 761–670 cm^−1^ are characteristic of albite, reflecting the contribution of the silicate/aluminosilicate fraction [38,39,40,41,42,43]. O–H groups are also observed in all three mixtures at 3428 cm^−1^, associated with water present in the mixtures. Additionally, organic C–H bands are detected in the range of 2953–2603 cm^−1^ and additional bands at 1843–1594 cm^−1^ [38,39,40,41,42,43], which increase in the order C1.1, C1.2, and C1.3. This confirms the incorporation of the organic fraction from the sludge into the base mixtures. A clear predominance of carbonate and aluminosilicate structures is established, which, further in the study serve as a reference basis for subsequent sulfate changes after activation.

The FTIR spectra of the acid-activated mixtures (C1.1.1, C1.2.1, and C1.3.1) show significant changes compared to the non-activated mixtures, indicating the occurrence of mineral transformations as a result of acid activation. In all samples, a clearly defined absorption band is observed around 1131–1136 cm^−1^, which corresponds to asymmetric stretching vibrations of SO_4_^2−^ groups and is characteristic of calcium sulfate, including bassanite (CaSO_4_·0.5H_2_O). The confirmation of sulfate groups is supported by the presence of bands at 597–599 cm^−1^ and 452 cm^−1^ [38,39,40,41,42]. These results are consistent with the XRD analysis, which shows the formation of bassanite, and confirm the effective sulfation of calcium-containing phases.

The stretching and bending vibrations of O–H groups appear at 3400–3540 cm^−1^ and around 1625–1685 cm^−1^, indicating the presence of structurally bound water and the formation of hydrated mineral phases [38,39,40,41,42]. This is characteristic of calcium sulfate hydrates and indicates the development of a stable hydrated mineral structure.

The presence of bands at approximately 750–761 cm^−1^, 659–665 cm^−1^, and 1392–1397 cm^−1^ corresponds to vibrations associated with aluminosilicate structures, including Na(AlSi_3_O_8_) and other silicate components [38,39,40,41,42]. This indicates the participation of silicate and aluminosilicate phases in the mineral matrix.

The observed spectral characteristics indicate substantial mineralogical transformation and structural evolution of the mixtures as a result of acid activation.

The FTIR spectra of the zeolite-modified mixtures (C1.1.3, C1.2.3, and C1.3.3) show, in all samples, an intense absorption band at 1126–1131 cm^−1^, corresponding to stretching vibrations of sulfate groups (SO_4_^2−^), confirming the incorporation of sulfate phases as a result of acid activation and the presence of calcium sulfate, including bassanite (CaSO_4_·0.5H_2_O). The presence of vibrations at 597–599 cm^−1^ and 452–453 cm^−1^ further confirms the formation of sulfate phases. As in the acid-treated mixtures, bands are observed in the region of 3405–3542 cm^−1^ and around 1625–1694 cm^−1^, corresponding to hydroxyl groups (O–H), indicating the presence of structurally bound water. Particularly characteristic of the modified mixtures are clearly defined bands at approximately 670 cm^−1^, 739–766 cm^−1^, and 1376–1377 cm^−1^, which are associated with the presence of aluminosilicate structures, including Na(AlSi_3_O_8_) and zeolite components. These spectral features indicate effective structural integration of zeolite into the mineral matrix [38,39,40,41,42]. The presence of a band around 1427–1429 cm^−1^, especially clearly expressed in mixtures C1.2.3 and C1.3.3, corresponds to carbonate groups, indicating partial preservation or reformation of calcium carbonate phases.

### 3.4. Microstructural Characterization (SEM–EDS)

#### 3.4.1. Microstructural Characterization of Raw Materials

The analysis confirms that FARP has a calcium-dominant system with a carbonate mineral matrix, and the presence of silicon (6.08%) is also confirmed. Carbon is present mainly in carbonate form rather than as organic carbon. The overall composition confirms and classifies FARP as a mineral system with a structural framework capable of inducing transformations in the mixtures.

**Figure 5:**
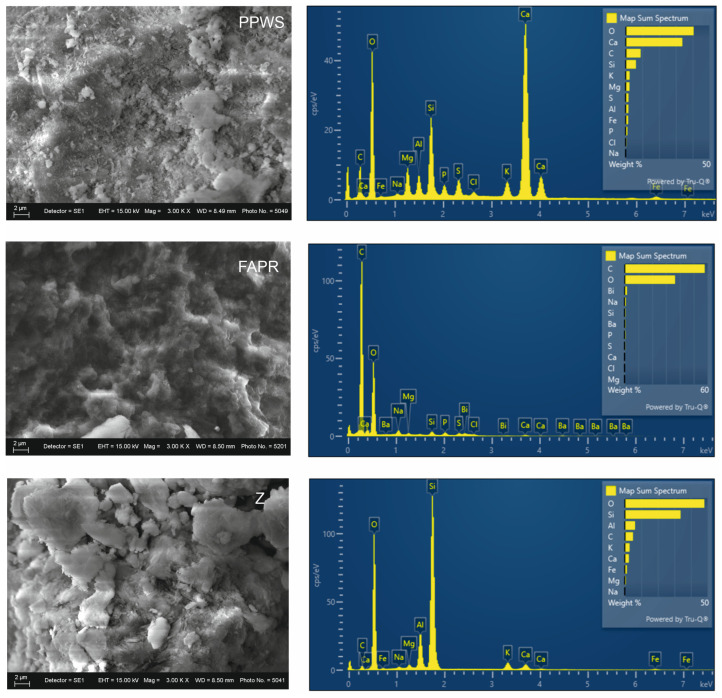
SEM-EDS analysis of raw materials.

SEM images show in Figure 5 irregularly shaped particles and aggregates, as well as particles with uneven surfaces. The morphology indicates a typical structure with a dominant mineral phase, characteristic of biomass ash. For PPWS, SEM images show amorphous aggregates and agglomerated particles, as well as irregular, uneven structures with porous morphology. Well-defined crystals are not observed, which is typical for wastewater sludge with high organic matter content. EDS analysis confirms the strongly organic character of the waste, with carbon content of 57.87% and oxygen content of 36.44%, along with low concentrations of calcium, silicon, and phosphorus. The results confirm that the contribution of PPWS is associated primarily with the amorphous organic matrix.

#### 3.4.2. Mixtures

**Figure 6:**
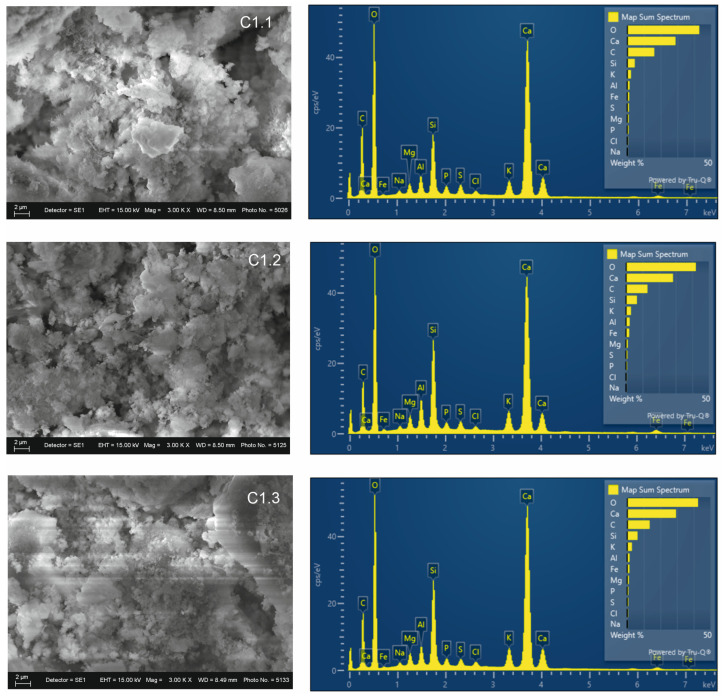
SEM-EDS analysis of binary mixtures.

In the binary mixtures, EDS analysis (Figure 6) shows calcium as the dominant element, with varying amounts of silicon: C 1.1–4.38%, C 1.2–6.55%, and C 1.3–6.17%, with C 1.2 having the highest aluminosilicate contribution among the three mixtures. The organic contribution is monitored through carbon content, which is highest in C 1.1 and lowest in C 1.2. SEM analysis of the non-activated mixtures shows morphological similarity to the raw materials. Agglomerates are present, with varying degrees of surface roughness and porosity.

**Figure 7:**
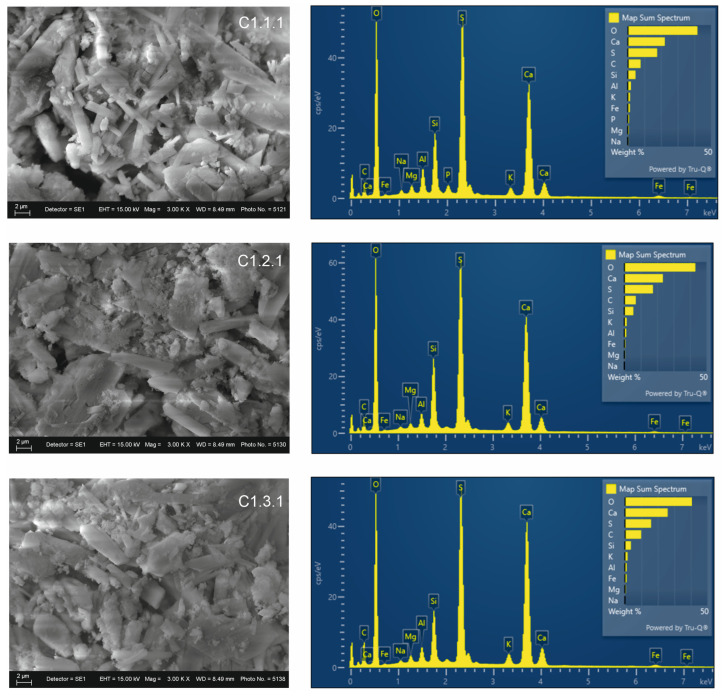
SEM-EDS analysis of acid treated mixtures.

SEM–EDS analysis of the acid-treated mixtures (Figure 7) confirms the increase in sulfur content and the formation of Ca–S structures. This results in partial redistribution and formation of new phases. Carbon content decreases, and the mineral phase becomes more clearly distinguishable. The images show a denser structure, with reduced porosity compared to the previous mixtures. A more homogeneous structure and finer particle dispersion are observed.

**Figure 8:**
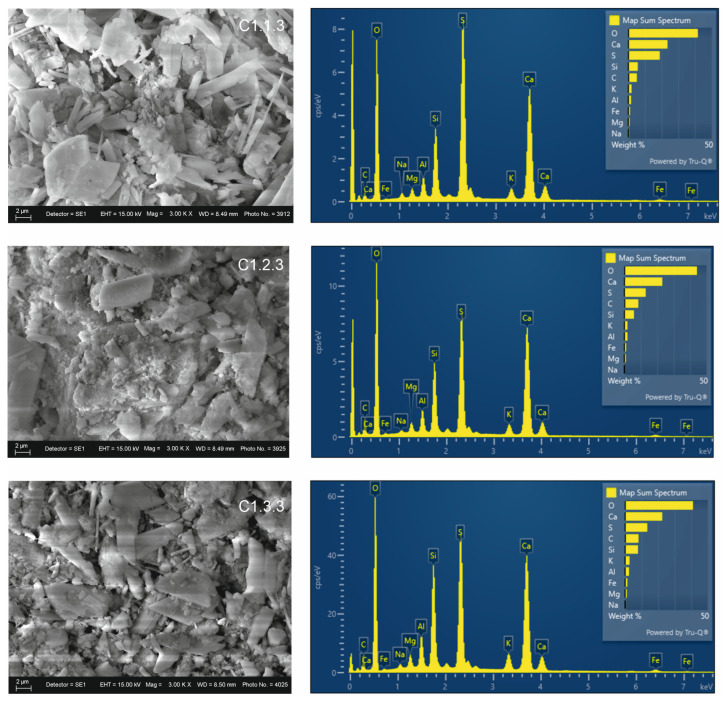
SEM-EDS analysis of zeolite modified mixtures.

In the zeolite-modified mixtures (Figure 8), calcium content remains approximately constant, with a strong contribution from aluminosilicates and sulfur. Silicon content is highest in mixture C 1.3.3 (7.7%), followed by C 1.2.3 (5.8%), and C 1.1.3 (5.6%). Carbon remains at low levels. The morphological effect of zeolite is expressed through the formation of a more homogeneous mineral structure and a well-developed microstructure.

SEM images of activated and modified mixtures show the predominance of needle-like/prismatic crystals forming aggregates and a more compact mineral matrix compared to the non-activated mixtures.

## 4. Discussion

### 4.1. Role of Raw Materials and Initial Structure of the Base Mixtures

The application of appropriate doses of wastewater treatment sludge from the pulp and paper industry into soil, after suitable treatment, contributes to increasing the content of carbon, nitrogen, and phosphorus, improving plant growth in various crops, and increasing pH [1,11,23]. On their own, they do not provide the expected increase in macroelements such as potassium and magnesium, which require prior enrichment before field application; however, in combination with biomass ash and other alkaline residues, sludge shows good results as a liming agent. ICP–OES analysis confirms the low concentration of calcium in the sludge, but it contains 2.47% sulfur, which is an essential element for the formation of amino acids, proteins, and enzymes in plant species. The amount of phosphorus would contribute to a good nutrient balance, and an additional contribution can be attributed to the presence of magnesium. The main functional role of PPWS is related to its organic fraction content. From a structural point of view, the sludge contributes with its amorphous phase and provides reactivity in the system by facilitating mineral integration.

On the other hand, biomass ash (FARP) increases pH due to the presence of carbonates, oxides, hydroxides, and silicates of calcium, magnesium, sodium, and potassium, and improves soil fertility. The high calcium content (30%) defines it as a major source for the formation of mineral phases. Calcium-rich biomass ash is widely recognized as a suitable precursor for the formation of stable mineral structures due to its ability to participate in hydration and pozzolanic reactions [3,29,30]. The positive effect on soils is associated with its liming effect, improvement of soil structure, stimulation of root system development, and enhancement of cation exchange capacity. Potassium is an essential micronutrient for plants, and its content in the ash contributes to regulating water balance and enzymatic activity, allowing plants to be more resistant to stress. Trace elements are present in significant amounts, supporting plant growth, photosynthesis, and improving plant resistance to diseases. The levels of toxic metals in both raw materials are below the limits established by environmental legislation [43].

Zeolite is characterized by high aluminum and silicon content, contributing aluminosilicate structural units capable of participating in the formation of the mineral network. Natural zeolites, especially clinoptilolite, are known as reactive aluminosilicate phases and nucleation centers that support the development and stabilization of the mineral structure [26,27,28]. The chemical composition of zeolite promotes cation exchange capacity, allowing retention of nutrients, their gradual release, and reduction of leaching.

The base mixtures (C1.1, C1.2, and C1.3) exhibit a clearly expressed organo-mineral structure, in which a significant amount of calcium (~30% in the original ash) is present, confirmed by both ICP-OES and SEM-EDS. Various types of ash capable of participating in mineral transformations and pozzolanic processes are widely described in the literature [3,25,29]. The concentrations of heavy metals in all raw materials are below the maximum permissible concentrations established by legislation [43]. However, some of them in the base mixtures are at borderline values (for example Cd—2 mg/kg), although they do not exceed the limits. All other elements are present at significantly lower concentrations, confirming the environmental suitability of the developed mixtures from the perspective of nutrient elements. The decrease in phosphorus is associated with Al/P phases and the formation of berlinite, which is related to the crystallization of phosphate phases. Aluminum phosphate is a poorly soluble form, which would contribute to the slow release of phosphorus for plant nutrition. Additionally, due to its chemical stability, it may fix heavy metals and reduce their mobility. It may also indirectly participate in the formation of stable mineral phases, leading to improved structural stability.

FTIR analysis shows clearly expressed carbonate bands (~2603–2944 cm^−1^ and ~1775–1843 cm^−1^), confirming the presence of calcium carbonate phases. Carbon from elemental analysis shows a moderate decrease, which is associated with transformation of the compositional ratios. Carbon stabilization increases the mineral components in the composite mixtures [29,30].

### 4.2. Chemical Behavior and Interaction Mechanisms

To better understand the observed transformations, the chemical behavior of each raw material is initially assessed on its own and then evaluated regarding interactions in native mixtures. Fly ash from plant residues (FAPR) is high in calcium and exhibits strong alkaline properties, accounting for its dominant function as the primary source of calcium in the system. Some XRD analysis confirmed the presence of calcite (CaCO_3_) and portlandite (Ca(OH)_2_) phases due to reactive forms of Ca that allows formation. These are preparatory stages of further reactions and create conditions for the formation of binding mineral structures. On the other hand, the PPWS is comprised primarily of an amorphous organic fraction and has a higher sulfur content. Here we present a reactive framework capable of undergoing structural changes upon chemical activation. PPWS can facilitate interactions with calcium-rich phases, which makes it highly reactive and helps in induced formation of new mineral structures. Zeolite is notably aluminosilicate frameworks, which makes them key agents in system modification, as they are characterized by high contents of Al and Si, ion-exchange capacity, and pozzolanic activity. As a nucleation substrate, it helps form stable aluminosilicate phases that aid in creating structural organization and ensuring the long-term stability of mixtures. In the absence of chemical activation, combining these materials produces a system dominated by calcium leading to carbonate and hydroxide phases. In this context, the reactivity is restricted and the structure is primarily controlled by CaCO_3_ and SiO_2_ phases. The addition of sulfuric acid leads to major changes in the system. Calcium is partially consumed via a reaction with sulphate ions, resulting in the development of Ca–S–O phases, mainly calcium sulfate hydrates (such as bassanite). This reaction accounts for the decrease in calcium content and concurrent increase in sulfur concentration noted for the activated mixtures. Calcium availability for carbonate formation is decreased as it is replaced in new interstitial fluid until the carbonate basin begins to fill. FTIR analysis further substantiates this transition with reactive groups shifting from SO_4_^2−^ dominating carbonate-based bands, reflecting a transition from carbonate-dominated to sulfate-dominated environments. Moreover, the variations of Si–O and Al–O vibration bands indicate partial reorganization in aluminosilicate framework. The presence of the additional Al and Si in zeolite-modified mixtures enhances further reaction between calcium with aluminosilicate components, producing C–A–S–H (calcium aluminosilicate hydrate) phases [37]. These phases are formed, which generally predominate in pozzolanic systems, have better structural stability than other phases. The decline of the Ca/Al ratio follows the element reorganization also supporting the change from calcium-dominated system to aluminosilicate-controlled matrix. Interpretation of XRD, FTIR and chemical analysis results highlights that the evolution of calcium-bearing systems can be observed in three main stages (i) calcium dominated carbonate system (ii) sulfate transformed intermediate structure after acid activation, and (iii) stabilized aluminosilicate matrix through incorporation of zeolites. This is indeed a step-by-step transition reflecting the formation of more complex and stable mineral structures. In summary, the resultant changes suggest that FAPR-PPWS-zeolite interactions are dominated by the equilibria of calcium availability versus sulfate formation and reactivity of aluminosilicates. The stable minerals generating through these processes like CaSO_4_ and these structural properties are improved through C–A–S–H suggest potential application in soil stabilization and construction related systems.

### 4.3. Mineral Transformation in Acid-Activated Mixtures (C1.1.1, C1.2.1, C1.3.1)

The results from XRD, FTIR, SEM, and elemental analysis consistently show significant mineral transformation after acid activation. With increasing sulfur content in the mixtures, a real sulfation process occurs, accompanied by a reduction of exchangeable calcium in the system and its fixation in a Ca–S–O structure. XRD analysis confirms the formation of calcium sulfate phases, including bassanite (CaSO_4_·0.5H_2_O). FTIR analysis confirms this observation through the appearance of sulfate absorption bands in the region of 1131–1136 cm^−1^, which confirms the incorporation of sulfate groups into the mineral matrix. Similar sulfation processes in calcium-rich systems have been widely documented in cementitious and pozzolanic systems [17,18,19,20].

SEM observations show the development of a more compact and homogeneous microstructure in the activated samples compared to the non-activated mixtures. Such structural evolution is characteristic of mineral systems subjected to chemical activation [19,20].

Phosphorus and potassium decrease after activation due to redistribution and the formation of new mineral matrices, and the presence of heavy metals is significantly reduced. This is related to the immobilization effect that occurs during the restructuring of sulfate and aluminosilicate phases in the mixtures.

After acid activation, the carbon content decreases further, which is also visible in the SEM images through the formation of a more compact matrix and the development of crystalline aggregates. This method enriches the inorganic phase and promotes recrystallization. The removal of the amorphous organic component leads to an increase in specific surface area, improved accessibility to aluminosilicates, and the formation of more stable mineral phases. This, in turn, leads to improved binding properties when applied in soil or cementitious mixtures.

### 4.4. Effect of Zeolite Modification on the Development of the Mineral Structure (C1.1.3, C1.2.3, C1.3.3)

The incorporation of zeolite leads to a further increase in the structural complexity and stability of the mineral matrix. The zeolite added to the mixtures additionally increases the aluminum content in the mixtures. Despite the fact that its amount in the mixtures is only 7%, it enables competing effects to occur in the mixtures and contributes to the formation of active centers [26,27,28]. FTIR analysis shows more pronounced Si–O–Si and Si–O–Al absorption bands in the zeolite-modified samples, confirming its increased contribution. SEM analysis shows a more homogeneous and well-integrated microstructure compared to the mixtures without zeolite. The aluminosilicate structure of zeolite contributes to the formation of stable mineral networks by providing reactive surfaces and structural stability. Similar effects have been reported in other mineral and cement systems containing zeolite [27,28].

In these mixtures, heavy metals are present at even lower concentrations due to the sorption capacity of zeolite and encapsulation within C–S–H/C–A–S–H structures, which is directly related to the concept of circular valorization. In the context of soil application, indicating favorable characteristics that may support their potential use as soil amendments. Since calcium is preserved in significant amounts (Ca ≈ 11.6–14.6%), sufficient to provide neutralization potential, CaSO_4_ and Ca-based silicate and aluminosilicate structures can act as sources of slowly released nutrients and improve the physicochemical properties of soil [26,27,28]. This contributes to improving nutrient efficiency and the stability of soil systems.

Zeolite also has favorable characteristics for carbon stabilization. This is supported by the presence of carbonate phases in the FTIR analysis, while SEM-EDS analysis shows a decrease in the relative content of organic carbon and the formation of a stable mineral matrix. This process contributes to reducing carbon mobility, which is essential for sustainable waste management and reducing the carbon footprint.

### 4.5. Significance for Pozzolanic Activity and Material Stability

The chemical composition, the reduction of calcium, and the observed mineral transformations such as aluminum stabilization indicate that the investigated mixtures possess characteristics typical of pozzolanic and cementitious systems. The presence of calcium, silicon, and aluminum creates favorable conditions for the formation of stable mineral structures [17,18,19,20].

The presence of amorphous components from the sludge and aluminosilicate components from the zeolite further supports structural development. This indicates that the obtained materials represent stable organo-mineral composites. XRD analysis shows the presence of anorthite, which plays a role in the mixtures due to its low to moderate pozzolanic activity, lower reactivity compared to the amorphous phase, and higher chemical stability. This mineral participates in the formation of cementitious systems such as C–A–S–H, stratlingite, and calcium aluminosilicate hydrate [5,11,16,17,18,19,28,29].

Such mineral systems have potential applications in construction, soil stabilization, and environmental technologies [21,25].

### 4.6. Confirmation of the Working Hypothesis

The obtained results demonstrate that waste materials such as biomass ash and wastewater sludge can be successfully transformed into stable mineral materials through appropriate chemical treatment. This is of significant importance for sustainable waste management and the valorization of secondary resources in accordance with the principles of the circular economy [5,14,21].

The results obtained from mixtures C1.1.1–C1.3.1 and C1.1.3–C1.3.3 confirm the working hypothesis that acid activation and zeolite modification lead to mineral transformation and the formation of a stable calcium–sulfate–aluminosilicate mineral matrix.

### 4.7. Compared to Existing Literature

The results are in accordance with findings from available literature on the use of biomass ash and sewage sludge as secondary resources for sustainable material systems. Biomass ash is high in calcium and several studies have shown it may act as a precursor of the development of mineral binding phases, such as calcium silicate hydrates (C–S–H) and calcium aluminosilicate hydrates (C–A–S–H) [6,17,18,19,20,24]. Indeed, the elevated calcium levels in this FARP (≈30%) are in agreement with previously reported data where calcium is identified as an important factor controlling phase formation and overall structure established throughout these ceramic materials. Well-known as well is the sewage sludge contribution to this pool of amorphous and reactive components [5,19,20,21]. Literature data states that sludge adds organic matter and nutrients, besides acting as a reactive matrix promoting transformation processes of minerals. This is in line with current results where PPWS shows low crystallinity and leads to generating organo-mineral structures. The formation of calcium sulfate phases after acid activation recorded in this work correspond to previous works describing sulfation processes in calcium-rich systems [15,16,17] The changes of CaCO_3_ and Ca(OH)_2_ to the respective Ca–S–O phases like that of bassanite, demonstrate the success of acid activation in altering mineral composition and improving structural durability. But similar mineral transformations have already been discussed in cementitious and pozzolanic systems treated by chemical activation. Moreover, the results from these studies provide further evidence for zeolite incorporation and its influence on aluminosilicate structure formation [26,27,28,30,31]. Zeolites are known for pozzolanicity, and functioning as crystallization nucleation sites facilitating stable mineral phase development. The increase in aluminum content and the presence of more complex aluminosilicate framework structures in the modified mixtures are consistent with previously reported effects for zeolite-modified systems. Also from the view of potential applications, results from this study are in accordance with literature reports which suggest that biomass ash [13,14] and sewage sludge [19,25] could be used as soil amendments or construction material components. Furthermore, their promising potential for soil applications is affirmed by the occurrence of macroelements (Ca, K, Mg and P), in addition to low concentrations on heavy metal. This finding is consistent with the formation of stable mineral phases and pozzolanic structures reported for supplementary cementitious materials and waste-derived binders [11,12,16,17,18]. It is worth mentioning that the majority of literature studies much have some application-related performance, such as mechanical strength or agronomical one. The conclusions in the current study are primarily based on physicochemical and mineralogical characterization. Thus, although the results are consistent with the existing research, further investigation is needed to fully evaluate the performance of these materials in practical applications.

## 5. Conclusions

As a management practice, the application of waste-derived materials for improving soil structure and creating structurally stable materials offers significant potential for climate change mitigation and provides additional environmental benefits; promoting the widespread implementation of climate mitigation practices should be a European priority. A significant effect of implementing resource valorization is the utilization of waste materials from the pulp and paper industry to support future environmental policy and a system of actions aimed at mitigating the causes of and adapting to anticipated climate change.

The obtained results demonstrate that waste materials from the pulp and paper industry, such as biomass ash and wastewater sludge, can be applied as soil amendments by supplying essential nutrients in stable structures that provide gradual nutrient release while simultaneously improving soil structure, particularly in reclaimed or degraded lands. The developed mixtures could be considered as promising candidates for use in cementitious systems, with potential economic and environmental benefits related to their stable structure and pozzolanic activity. In accordance with the principles of the circular economy, the use of waste materials represents a sustainable approach for resource recycling and waste reduction.

The obtained results indicate that the zeolite-modified mixtures C1.1.3, C1.2.3, and C1.3.3 demonstrate significant potential for application as functional soil amendments and construction materials. In this way, a closed resource loop is achieved, where industrial waste is transformed into valuable functional materials, supporting sustainable resource management and the concept of “from farm to table and back again.”

## Figures and Tables

**Table 1:** Composition of prepared mixtures (wt%).

Mixtures	PPWS (wt.%)	FAPR (wt.%)	H_2_SO_4_ (wt.%)	Z (wt.%)
C 1.1	75	25	0	0
C 1.2	59	41	0	0
C 1.3	67	33	0	0
C 1.1.1	67	23	10	0
C 1.2.1	52	38	10	0
C 1.3.1	60	30	10	0
C 1.1.3	56	27	10	7
C 1.2.3	48	35	10	7
C 1.3.3	55	28	10	7

Notes: PPWS—Pulp and Paper Wastewater Sludge; FARP—fly ash from plant residues; Z—Zelolite.

**Table 2:** Composition of raw materials.

Parameter	Unit	FARP	PPWS	Z
Ca	%	30.48	0.29	2.48
Al	%	1.20	0.02	4.02
Fe	%	1.05	0.02	0.48
K	%	1.11	0.02	0.93
Mg	%	0.83	0.03	0.44
P	%	0.41	0.10	0.004
S	%	0.06	2.47	0.01
Na	mg/kg	2838	984	3175
Zn	mg/kg	122	13	34
B	mg/kg	90	12	5
Ba	mg/kg	466	57	18
Cd	mg/kg	1	<0.1	<0.1
Co	mg/kg	4	<0.5	<0.5
Cr	mg/kg	25	4	<0.5
Cu	mg/kg	60	6	2
Mn	mg/kg	1617	391	479
Mo	mg/kg	1	<0.5	<0.5
Ni	mg/kg	19	<0.5	<0.5
Pb	mg/kg	19	<0.5	53
V	mg/kg	26	<1	2

**Table 3:** Chemical composition of the mixtures.

Parameters (Unit)	C 1.1	C 1.1.1	C 1.1.3	C 1.2	C 1.2.1	C 1.2.3	C 1.3	C 1.3.1	C 1.3.3
Ca (%)	30.21	13.2	11.6	31.31	24.44	13.68	33.32	23.83	14.6
Al (%)	1.12	0.70	0.90	1.24	0.91	1.31	1.20	0.87	1.21
Fe (%)	0.97	0.65	0.58	1.03	0.79	0.74	1.02	0.74	0.72
K (%)	1.03	0.78	0.79	1.08	0.90	0.91	1.13	0.88	0.88
Mg (%)	0.76	0.59	0.52	0.85	0.67	0.62	0.88	0.65	0.65
P (%)	0.53	0.36	0.25	0.50	0.38	0.26	0.56	0.40	0.30
S (%)	0.05	5.40	4.62	0.05	3.01	2.52	0.04	8.77	7.20
Na (mg/kg)	3662	3005	2894	3548	2866	2884	3648	2969	3006
Zn (mg/kg)	124	72	63	132	102	88	143	93	86
B (mg/kg)	91	67	54	95	77	64	100	73	70
Ba (mg/kg)	466	256	253	505	334	194	514	218	333
Cd (mg/kg)	2	0.98	0.86	2	1	0.99	2	1	1
Co (mg/kg)	4	3	2	4	3	3	4	3	3
Cr (mg/kg)	29	19	15	31	22	19	30	21	18
Cu (mg/kg)	61	45	36	70	47	43	71	46	43
Mn (mg/kg)	1894	1265	1090	1810	1473	1228	1951	1456	1321
Mo (mg/kg)	2	2	1	2	1	1	2	1	1
Ni (mg/kg)	19	11	9	21	14	12	21	13	12
Pb (mg/kg)	21	18	20	27	18	24	30	18	22
V (mg/kg)	21	14	11	23	17	15	22	16	15

**Table 4:** Elemental composition and distribution ratios in the mixtures.

Parameters	Nitrogen N, %	Carbon, C, %	Hydrogen H, %	C:H (Atomic)	C:N (Atomic)	C:S (Atomic)
C 1.1	0.27	10.74	0.95	0.95	46.39	573.44
C 1.1.1	0.28	9.46	1.12	0.71	39.40	4.68
C 1.1.3	0.23	8.73	0.98	0.75	44.26	5.04
C 1.2	0.28	11.04	1.01	0.92	45.98	589.46
C 1.2.1	0.25	9.23	1.55	0.50	43.06	8.19
C 1.2.3	0.22	10.76	1.28	0.71	57.04	11.40
C 1.3	0.19	9.36	1.18	0.67	57.45	624.69
C 1.3.1	0.27	8.46	0.82	0.87	36.54	2.58
C 1.3.3	0.23	9.65	1.35	0.60	48.93	3.58

## Data Availability

The original contributions presented in this study are included in the article. Further inquiries can be directed to the corresponding author.
